# Biomechanical evaluation of different screw fixation methods for Ogawa type I coracoid process base fracture

**DOI:** 10.1016/j.jseint.2025.101423

**Published:** 2025-12-02

**Authors:** Yiwen Tan, Zhao Tan, Hu Zhang, Fangfang Mou

**Affiliations:** a Department of Orthopaedics, Shuguang Hospital Affiliated to Shanghai University of Traditional Chinese Medicine, Shanghai, China; b Anatomy Teaching and Research Section, Shanghai University of Traditional Chinese Medicine, Shanghai, China

**Keywords:** Coracoid process, Fracture, Biomechanical experiment, Screw fixation, Internal fixation, In-out-in

## Abstract

**Background:**

The internal fixation of Ogawa type I coracoid fractures is relatively difficult, and there is no consensus on the fixation method, which is also prone to screw cutout. This study aims to evaluate the biomechanical properties of 5 different screw internal fixation methods for Ogawa type I coracoid process base fracture through biomechanical experiments.

**Hypothesis:**

The biomechanical effects of the 5 fixation methods are different.

**Methods:**

Fifteen fresh adult scapula specimens were randomly selected to create models of Ogawa type I coracoid process base fracture. Five fixation methods were used: single hollow screw fixation entirely within the bone (M1), single hollow screw fixation partially exiting below the vertical part of the coracoid process (M2), single hollow screw fixation partially exiting above the vertical part of the coracoid process and extending to the scapular spine (M3), dual hollow screw fixation entirely within the bone (M4), and dual hollow screw fixation combining M1+M3 (M5). All specimens were randomly divided into 5 groups of 3, each corresponding to a fixation method. Biomechanical experiments of compression, tension, and rotation were conducted to assess the stability of each fixation method.

**Results:**

There was no significant difference in bone mineral density among the groups (*P* = .873). At the compression end point, M2 sustained the lowest force and M4 the highest, with the ranking M2 < M3 < M1 < M5 < M4; the intergroup difference was statistically significant (*P* = .022). At the tension end point, M3 sustained the lowest force and M5 the highest, with the ranking M3 < M2 < M1 < M4 < M5; the intergroup difference was statistically significant (*P* = .019). At the torsion end point, M2 showed the lowest torque and M5 the highest, with the ranking M2 < M3 < M1 < M4 < M5; the intergroup difference was statistically significant (*P* = .032).

**Conclusion:**

The dual-screw fixation methods (M4 and M5) demonstrated the best stability in treating Ogawa type I coracoid process base fracture, with no significant difference between M5 and M4. For single-screw fixation, the entirely intraosseous method (M1) was more stable than the “in-out-in” methods, and the mode with partial downward exit (M2) should be avoided.

The coracoid process is a bony projection extending from the upper margin of the scapular neck. Its vertical part extends forward and upward, then sharply bends to protrude forward and laterally, forming the horizontal part. The coracoid process serves as the attachment site for the coracobrachialis muscle, the short head of the biceps brachii muscle (forming the conjointed tendon), and the pectoralis minor muscle. It is also connected to the transverse scapular ligament, the coracoclavicular ligament, the coracohumeral ligament, and the coracobrachialis ligament, making it a key structure in maintaining the connection between the clavicle and the scapula.^1^^,^^17^ Although the incidence of coracoid process fractures is not high,^7^ its unique and complex anatomical structure determines the difficulty of treatment. If the fracture is not managed properly, it can easily lead to shoulder dysfunction, severely affecting the patient's quality of life.^10^

The classic clinical classifications for coracoid process fractures are the Ogawa classification^18^ and the Eyres classification.^8^ Compared with the Ogawa type II fractures that involve the horizontal part, the Ogawa type I fractures that involve the vertical part are more common (approximately 77%),^17^ and the conventional surgical method is to fix the fracture through the vertical part of the coracoid process to the scapular neck using screws,^16^ typically with 1 to 2 screws. The thickness of the base of the coracoid process (approximately 10 mm) is much smaller than its width (approximately 23-26 mm).^6^^,^^9^ Therefore, when using screws with a diameter of 4.0 or 4.5 mm for fixation, it is easier for the screws to exit above or below the vertical part of the coracoid process, before re-entry into the scapular neck, creating an “in-out-in” fixation. Although the “in-out-in” technique has been applied in the fixation of other fractures with good results,^3^^,^^5^^,^^12^ there are no relevant studies on its application in coracoid process fractures. Therefore, we compared 5 different screw fixation techniques for Ogawa type I coracoid process base fracture, simulating fixation outcomes commonly encountered in clinical practice. Biomechanical testing was used to evaluate the effectiveness of each method. We hypothesized that double-screw fixation would provide superior stability, and that the “in-out-in” technique would also yield satisfactory results.

## Materials and methods

### Specimen preparation

**Figure 1:**
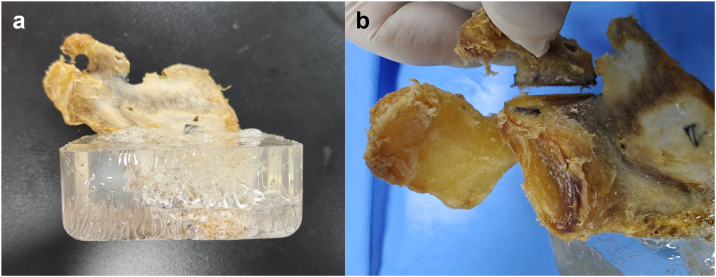
Preparation of the specimens and fracture models. (**a**) The scapula specimen fixed in epoxy resin. (**b**) The prepared model of Ogawa type I coracoid process base fracture.

Fifteen fresh adult scapula specimens, including 8 right and 7 left, were provided by the Anatomy Teaching and Research Section of Shanghai University of Traditional Chinese Medicine. The muscles and connective tissues were removed from the specimens to obtain 15 complete osseous scapula specimens. The specimens were stored in a low-temperature freezer at −30°C and thawed gradually before the experiment. The scapulae were placed in a mold with the osteotomy surface facing upward, and epoxy resin was poured in to form a base of 10 × 10 × 5 cm^3^ after solidification, with the scapulae firmly fixed within the base. A transverse osteotomy was performed at the base of the coracoid process using an oscillating saw to create a model of Ogawa type I coracoid process base fracture (Fig. 1).

### Five fixation methods for the coracoid process fractures

**Figure 2:**
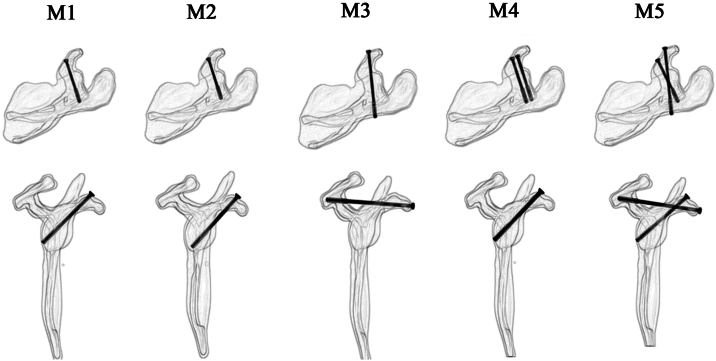
Five fixation methods for coracoid process base fractures.

The coracoid process base fracture was fixed using hollow screws. The screws used were 4.0 mm in diameter, partially-threaded hollow titanium screws provided by Changzhou Dingjian Medical Devices Co., Ltd. (Changzhou, China). The fixation schemes were divided into the following 5 groups (Fig. 2):

1) Single hollow screw fixation entirely within the bone (M1): A single screw is inserted along the long axis of the vertical part of the coracoid process, with the entire screw remaining within the bone throughout the vertical part and the distal end fixed to the posterior-inferior aspect of the scapular neck.
2) Single hollow screw fixation partially exiting below the vertical part of the coracoid process (M2): A single screw enters from the surface of the coracoid process, exits the cortex below the vertical part and travels for a short distance before re-entering the bone of the scapular neck. This simulates the “in-out-in” fixation method in the downward direction.
3) Single hollow screw fixation partially exiting above the vertical part of the coracoid process and fixed to the scapular spine (M3): A single screw enters from the surface of the coracoid process, exits the cortex above the vertical part and travels for a short distance before being fixed into the bone of the scapular spine. This simulates the “in-out-in” fixation method in the upward direction.
4) Dual hollow screw fixation entirely within the bone (M4): Two hollow screws are both fixed within the bone of the vertical part of the coracoid process and extend to the bone of the scapular neck.
5) Dual hollow screw fixation combining entirely within the bone and partially exiting above (M5): This is a combination of M1 and M3.

### Biomechanical experiments

Biomechanical experiments were conducted using the Instron ElectroPlus E3000 system (Illinois Tool Works Inc., Glenview, IL, USA). The specimens, after screw fixation, were securely mounted on a fixture below the frame, with the coracoid fracture fragment positioned directly above and the fracture surface oriented horizontally upward. Loads of compression, tension, and rotation were applied to the fracture fragment to evaluate the biomechanical efficacy of different screw fixation methods. Prior to the formal experiment, a preload of 50 N was applied multiple times to eliminate the viscoelastic effects such as bone relaxation and creep.

**Figure 3:**
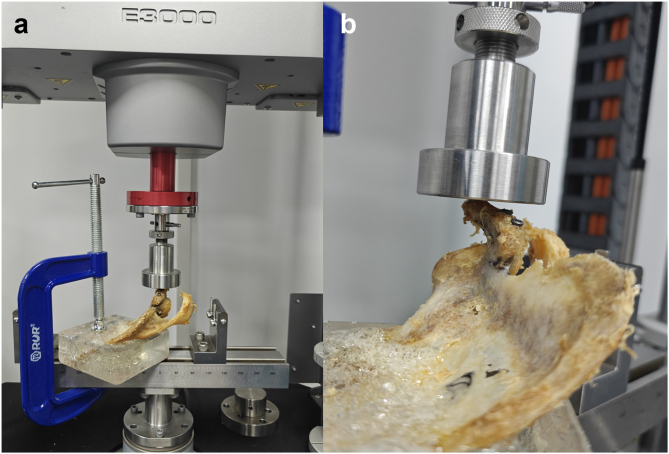
Compression testing. (**a**) Placement of the specimen. (**b**) The point of compression force is located above the tip of the coracoid process.

Compression: To simulate the daily forces exerted on the coracoid process by the conjointed tendon and the pectoralis minor tendon, which primarily act on the tip and the horizontal part of the coracoid process, the testing module was placed directly above and in contact with the bone surface at the tip of the coracoid process, rather than directly above the fracture site (Fig. 3). A compression rate of 2 mm/min was applied, and the test was terminated when the compression displacement exceeded 3 mm or the applied force exceeded 500 N. The force required to achieve different levels of compression displacement was recorded.

Tension: The upper testing module and the coracoid bone fragment were fixed with a clip to ensure they were aligned on the same axis. Axial tension was applied at a rate of 2 mm/min, and the test was terminated when the tensile displacement exceeded 3 mm or the applied force exceeded 500 N. The force required to achieve different levels of tensile displacement was recorded.

**Figure 4:**
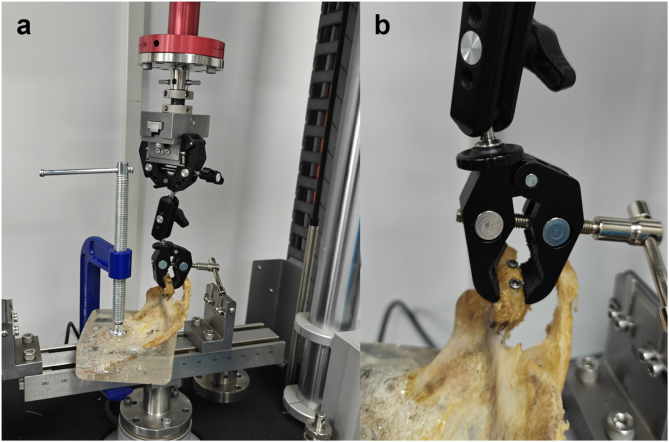
Tension and rotation testing. (**a**) Placement of the specimen. (**b**) Fixation position of the clip.

Rotation: The fixation method was the same as that for tension testing (Fig. 4). Axial counterclockwise rotation was performed at a speed of 10°/min, and the test was terminated when the rotation angle reached 10°. The torque required to achieve different rotation angles was recorded.

### Experimental Design and Data Statistics

Fifteen specimens were randomly divided into 5 groups of 3, each corresponding to a fixation method, and were labeled M1–M5. Every specimen underwent compression, tension, and rotation tests in sequence. After each test, the clip and test module were loosened, the screws retightened, and the next test performed. Consequently, each fixation method yielded 3 sets of results for compression, tension, and rotation. Because the specimen provider was unable to supply age data, age information is missing for all specimens. To control for individual biological variability, we therefore added a bone-density measurement for every specimen. Specifically, a 1 cm^3^ bone block was harvested from the identical acromial region of each specimen, to avoid any influence on the coracoid process experiment, and its volumetric bone mineral density (g/cm^3^) was determined with high-resolution micro–computed tomograpgy (SkyScan 1,172 desktop system; Bruker, Kontich, Belgium). After the specimens had been randomly assigned to the test groups, the bone mineral density values were compared across groups to check for any intergroup differences.

Descriptive statistics (mean and standard deviation) were used to summarize the data. The Kruskal-Wallis test for independent samples was employed to compare the different fixation methods under compression, tension, and rotation, and also to assess differences in bone mineral density among groups. The significance threshold was set at *P* < .05. Data analysis was conducted using SPSS software (version 24; IBM Corp., Armonk, NY, USA). Force-displacement curves for compression and tension, as well as torque-angle curves for rotation, were plotted for the different fixation methods.

## Results

The average bone mineral density of the 15 specimens was 0.576 ± 0.086 g/cm^3^. No statistically significant difference was found among the 5 groups (*P* = .873).

**Figure 5:**
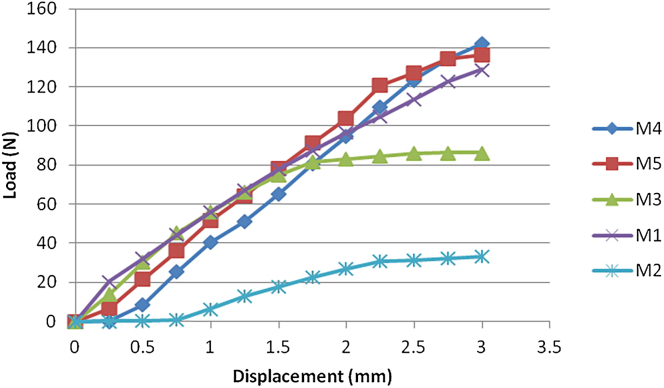
Force-displacement curves for compression testing of the 5 fixation methods.

Compression: When the compression displacement reached 3 mm, the force experienced by all 5 fixation methods did not exceed 150 N. The forces were as follows: M2 = 33.00 ± 8.70 N (smallest), M3 = 86.22 ± 20.00 N, M1 = 128.78 ± 2.93 N, M5 = 136.42 ± 14.64 N, and M4 = 142.14 ± 6.19 N. There were significant differences among the groups (*P* = .017). The order was M2 < M3 < M1 < M5 < M4. The force-displacement curves for compression are shown in Figure 5.

**Figure 6:**
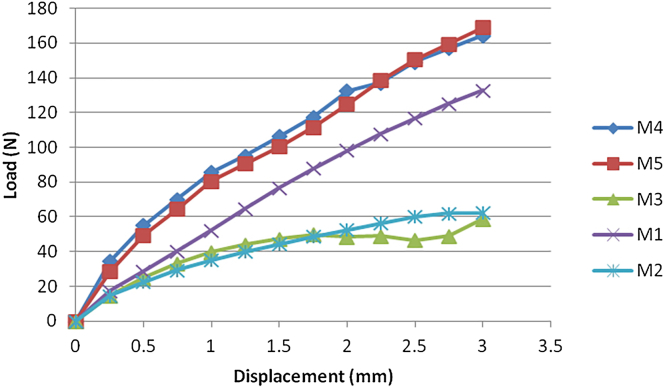
Force-displacement curves for tension testing of the 5 fixation methods.

Tension: When the tensile displacement reached 3 mm, the force experienced by all 5 fixation methods did not exceed 200 N. The forces were as follows: M3 = 58.66 ± 8.29 N (smallest), M2 = 62.19 ± 3.54 N, M1 = 132.81 ± 4.36 N, M4 = 164.39 ± 8.26 N, and M5 = 169.06 ± 24.18 N. There were significant differences among the groups (*P* = .016). The order was M3 < M2 < M1 < M4 < M5. The force-displacement curves for tension are shown in Figure 6.

**Figure 7:**
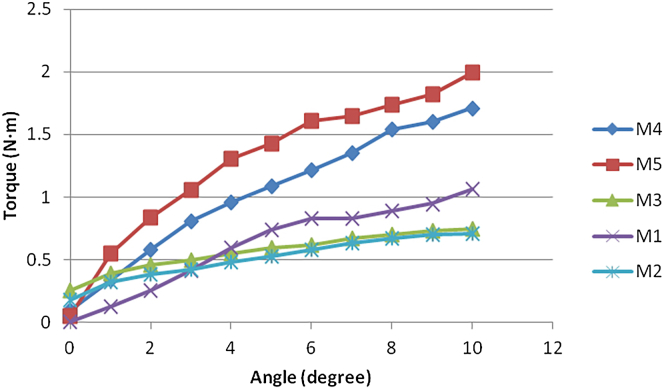
Torque-angle curves for rotation testing of the 5 fixation methods.

Rotation: When the rotation angle reached 10°, the torque experienced by all 5 fixation methods did not exceed 2.00 N m. There were significant differences among the groups (*P* = .032). The torques were as follows: M2 = 0.71 ± 0.19 N m (smallest), M3 = 0.75 ± 0.31 N m, M1 = 1.07 ± 0.61 N m, M4 = 1.71 ± 0.02 N m, and M5 = 1.99 ± 0.33 N m. The order was M2 < M3 < M1 < M4 < M5. The torque-angle curves for rotation are shown in Figure 7.

## Discussion

This study is the first to use biomechanical experiments to evaluate the efficacy of different internal fixation methods for the coracoid process base fractures, especially comparing the “in-out-in” fixation method. We found that the dual-screw fixation methods demonstrated the best stability and can be considered the preferred clinical treatment options, with no significant difference between M5 and M4. For single-screw fixation, the entirely intraosseous method (M1) was the most stable and the partially downward exiting method (M2) should be avoided.

Previous statistics have shown that scapular fractures account for 0.4%-1% of all fractures, and coracoid process fractures represent only 0%-8% of scapular fractures. However, with the advancement of imaging technology and increased awareness of fracture, the detection rate of coracoid process fractures has significantly increased.^17^ Currently, there are no definitive guidelines for the treatment of coracoid process fractures. It is generally believed that unstable, significantly displaced coracoid process fractures, as well as those involving 2 or more parts of the superior shoulder suspensory complex (SSSC), require surgical treatment.^10^^,^^24^ Conservative treatment yields satisfactory outcomes for Ogawa type II fractures and selected isolated type I fractures without concomitant SSSC disruption. In contrast, 69%-76% of Ogawa type I fractures are associated with SSSC injury, and these patients are better suited to surgical management.^17^^,^^7^

For Ogawa type I coracoid process fractures involving the base, exposure is extremely challenging due to the deep location of the fracture and the presence of many important structures around the coracoid process. Indirect reduction and screw placement guidance are provided by palpation with the fingertips, as direct visualization is not possible, adding a certain level of difficulty. Similarly, intraoperative fluoroscopy is also difficult to achieve satisfactory results due to the irregular anatomical shape of the coracoid process and limitations of patient positioning. Trikt et al^23^ and Sun et al^21^ used a projection view directly aligned with the vertical part of the coracoid process, known as the “Coracoid Tunnel View”, to guide screw placement. Bhatia proposed intraoperative fluoroscopy in the upper and lower pillar positions,^2^ and the classic scapular Y-view^13^ can also be used. However, due to patient positioning and other factors during surgery, it is difficult to obtain satisfactory fluoroscopy with the above views, which can lead to deviations in the judgment of the position of internal fixation. In addition to screws that remain entirely within the bone of the vertical part of the coracoid process, it is also relatively easy for screws to exit above or below the vertical part, forming an “in-out-in” fixation. If the “in-out-in” fixation method can achieve results similar to conventional fixation methods, then the difficulty of surgery and the requirements for fixation position can be appropriately reduced. Therefore, we designed this biomechanical study to evaluate the efficacy of different fixation methods, particularly the “in-out-in” technique, for the coracoid process base fractures.

Biomechanical experiments related to the coracoid process have mostly focused on coracoclavicular ligament reconstruction^4^^,^^14^ and the Latarjet procedure for treating shoulder instability,^15^^,^^19^ while biomechanical studies on fixation methods for coracoid process fractures are relatively rare. In our study, if a greater force is required to achieve the same compression or tensile displacement, it indicates that the fixation method is more stable. Similarly, if a greater torque is required to achieve the same rotation angle, it suggests that the fixation method has stronger and more stable resistance to rotation. Referring to the force direction of the coracoid process calculated by Seth et al^20^ in their scapular model, the dynamic force direction of the coracoid process is the resultant force direction of the conjointed tendon and pectoralis minor, with the point of action located near the tip of the horizontal part of the coracoid process. Therefore, in our compression experiment, the point of force application was placed directly above the tip of the coracoid process, rather than directly above the fracture site.

The results show that under compressive force, the dual-screw fixation methods (M4 and M5) and the single-screw method entirely within the bone (M1) demonstrated the best stability. Among the single-screw “in-out-in” fixation methods, M3 was superior to M2. The speculated reason is that for M2, both the fixation screw and the point of force application are on the same side of the fracture. During compression, the fracture ends tend to separate with the screw acting as a fulcrum, similar to a “seesaw”. In contrast, for M3, the fixation screw and the point of force application are on opposite sides of the fracture. When force is applied, the screw on the opposite side exerts a counteracting force, creating a compressive force at the fracture site, similar to the “guy ropes of a tent".

Under axial tensile force, the dual-screw fixation methods (M4 and M5) remained the most stable, with M1 being slightly less stable but still significantly better than M3 and M2. There was no significant difference between M2 and M3. This indicates that during resistance to tension, screws entirely within the bone play a major counteracting role, while the "in-out-in" screws provide a weaker counteracting force.

Under axial rotational force, the dual-screw methods (M4 and M5) still showed better performance than the single-screw methods (M1, M3, and M2). In summary, the dual-screw fixation methods demonstrated the best fixation effect, with no significant difference between the dual-screw method with partial upward exit and the dual-screw method entirely within the bone. For single-screw fixation, the entirely intraosseous method should be preferred, especially avoiding the “in-out-in” mode with partial downward exit. This is consistent with the clinical results of Tan et al,^22^ whose modified dual-screw method did not achieve the “in-out-in” effect with upward exit but was inserted in the direction of the scapular spine with more bone mass.

According to the research data of Montgomery et al,^15^ when the elbow is flexed at 90° and the hand is holding no weight, the combined force of the coracoclavicular ligament is between 148 and 242 N. When holding a 2-kg weight, it is between 200 and 340 N. Also, the study by Heilmann et al^11^ showed that the load to failure of an intact coracoid process is 428.2 N. In our experiments, the force at which the tension tests were terminated did not exceed 200 N, and the force at which the compression tests were terminated did not exceed 150 N. This means that none of the fixation methods were able to restore the mechanical strength of an intact coracoid process. Therefore, postoperative immobilization and gravity-resistant fixation are still very necessary. We recommend strict avoidance of weight-bearing and shoulder movement within 4 weeks after surgery. Range-of-motion exercises for the shoulder may begin at 6 weeks, but loaded biceps contractions should be avoided until 8 weeks postoperatively.

The limitations of this study are as follows: (i) due to the limited number of specimens and the relatively large number of fixation methods, only 3 specimens could be assigned to each group. This small sample size may introduce bias and diminish the reliability of both the experimental findings and the statistical analyses. (ii) Although interspecimen bone mineral density differences were found to be negligible, the unknown age of the specimens could restrict the generalizability of the result. For instance, if the donors were elderly, the outcomes might not be applicable to younger patients. (iii) The influence of surrounding soft tissues and ligaments was not considered. (iv) We used the clinically most commonly used 4.0 mm diameter partially threaded hollow screws as the fixation device, but different fixation devices, including screws of different diameters and pitches, may affect the results. (v) In addition, fractures were created with an oscillating saw to standardize location and pattern for comparative purposes. However, this produces a flat fracture surface devoid of the interdigitating comminution which is commonly seen clinically, so the model does not fully replicate real-world fracture morphology or the loading conditions at the fracture site.

## Conclusion

This study evaluated the efficacy of 5 different screw internal fixation methods for treating Ogawa type I coracoid process base fractures through biomechanical experiments. The dual-screw fixation methods (M4 and M5) demonstrated the best stability in all tests, with no significant difference between M5 and M4. For single-screw fixation, the entirely intraosseous method (M1) was more stable than the “in-out-in” methods, and the mode with partial downward exit (M2) should be avoided.

## Disclaimers

Funding: This research did not receive any specific grant from funding agencies in the public, commercial, or not-for-profit sectors.

Conflicts of interest: The authors declare that they have no competing interests. The authors, their immediate families, and any research foundation with which they are affiliated have not received any financial payments or other benefits from any commercial entity related to the subject of this article.
